# Forward Outlook on Oral Health: The American Association of Public Health Dentistry’s 2025 Research Agenda for Dental Public Health

**DOI:** 10.1111/jphd.70005

**Published:** 2025-08-02

**Authors:** Sepideh Banava, Julie C. Reynolds, Shillpa Naavaal, Julie Frantsve-Hawley, Mina Habibian, Christina Murphey

**Affiliations:** 1 Oral Epidemiology and Dental Public Health Division, Department of Preventive and Restorative Dental Sciences, School of Dentistry, University of California San Francisco, San Francisco, California, USA; 2 University of Iowa College of Dentistry, Preventive and Community Dentistry, Iowa City, Iowa, USA; 3 Pediatric Dentistry and Dental Public Health and Policy, School of Dentistry, Virginia Commonwealth University, Richmond, Virginia, USA; 4 Temple University Maurice H. Kornberg School of Dentistry, Philadelphia, Pennsylvania, USA; 5 Herman Ostrow School of Dentistry, University of Southern California, Los Angeles, California, USA; 6 College of Nursing and Health Sciences, Texas A&M University, Corpus Christi, Texas, USA

**Keywords:** AAPHD, dental public health, research agenda, research priorities

## Abstract

**Objectives:**

Dental public health (DPH) research is crucial in improving and promoting community oral health by generating and translating new knowledge into practice. Since the last AAPHD research agenda in 1992, the DPH field has made significant advancements. This research agenda aims to outline key research areas in DPH, including priority topics and evidence gaps in our scientific understanding of factors influencing population oral health and oral health equity.

**Methods:**

A workgroup under the AAPHD Council on Scientific Information (CSI) surveyed 503 AAPHD members in September 2021 to gather feedback on seven draft research objectives. The survey used a Likert scale to assess agreement and included open-ended suggestions.

**Results:**

A total of 115 AAPHD members responded to the survey, resulting in a response rate of about 23%. Six of the seven objectives were agreed or strongly agreed upon by respondents as important areas of focus for DPH research. The final research agenda includes six key areas: oral health policy and legislation, delivery system innovations and care models, oral health workforce, DPH education, social determinants of health, and epidemiology and surveillance systems. The research agenda describes advancements and gaps in these areas, existing evidence, example priority topics, and research questions.

**Conclusions:**

The AAPHD encourages DPH partners, including federal agencies, DPH organizations, foundations, and dental associations, to advocate for, support, use, and fund high-quality DPH research. This support is crucial for translating emerging evidence into oral health improvements and equity across the diverse US communities.

## 1 | Introduction

Dental Public Health (DPH) is “the science and art of preventing and controlling dental diseases and promoting dental health through organized community efforts. It is that form of dental practice that serves the community as a patient rather than the individual” [1]. Applied DPH research is an essential mechanism to promote oral health through the creation and translation of new knowledge into practice. Research agendas create a shared vision for researchers in a field of study, including gaps, priority areas, and opportunities for collaboration. The most recent research agenda for the DPH discipline was developed and published in 1992 in a Special Issue of the *Journal of Public Health Dentistry* as a result of a major joint effort between the American Association of Public Health Dentistry (AAPHD) and the American Public Health Association (APHA) Oral Health Section, along with the support of numerous other professional organizations [2]. That issue described research priorities and gaps in four main categories: epidemiology, prevention, health education and health promotion, and health services. Within epidemiology, the agenda called to improve oral health and disease measures, and to improve surveillance studies in disease-specific areas (e.g., caries, oral cancer) and for high-risk populations (e.g., individuals with special needs, people without housing) [3]. Priority areas within prevention included fluoride intake and sealant use, among others [4]. In health education and promotion priority, the agenda highlighted the role of social factors in influencing oral health behaviors, health literacy, and communication between providers and patients among other key areas [5]. The health services research component noted a general lack of funding and support for dental health services research, as well as the need for measures of oral health status and significant progress in research on access to care and quality of care [6].

DPH has seen immense progress in many of these research areas in the last 30 years, particularly in the areas of oral disease prevention, health services research, development of oral health measures, access to care, and the social determinants of oral health. Yet there are many areas that continue to remain critical gaps, such as a need for multidisciplinary and participatory approaches in research and program implementation, and development and evaluation of policies and interventions that support improvements in oral disease and dental care access for all. This progress, along with the evolution of its associated scientific evidence, priorities related to barriers and facilitators of oral health care and oral health equity, demographic changes, and the emergence of new technology, all necessitate a revision and update of the 1992 research agenda to provide a vision for DPH research in the 21st century.

To that end, with the support of the AAPHD Board of Directors, the authors have developed this updated agenda. This agenda will be regularly reviewed and revised as determined by AAPHD, to ensure it remains current and relevant. The process of regular updates will allow for the incorporation of new research findings, emerging technologies, changes in DPH priorities, and DPH trends. This ensures that the agenda continues to guide and inform efforts to improve community oral health and achieve oral health equity.

As noted in the 1992 agenda, the scope of research in DPH is enormous. Therefore, this agenda includes key, broad-based topic areas within the scope of DPH, but does not encompass all possible issues that could be relevant to DPH. This research agenda aims to highlight key research areas in DPH, including priority topics and evidence gaps in the field of population oral health. The target audience for this research agenda includes anyone who conducts or uses DPH-related research, including researchers, educators, advocates, funders, practitioners, policymakers, and the public.

The specific objectives of this research agenda, which were adapted from the 1992 AAPHD agenda [2], include:

1. Identify and list key research areas and priority topics in DPH research
2. Raise awareness about priority topics in DPH research
3. Stimulate and advance innovative research on key topics using methodological approaches particularly suited to advance knowledge and practice of DPH.
4. Enhance communication and collaboration to identify funding opportunities, support the development of new funding avenues, foster collaboration, and facilitate the translation of DPH-related research into practical applications

## 2 | Methods

The development of this research agenda was spearheaded by a dedicated workgroup under the direction of the AAPHD Council on Scientific Information (CSI). The workgroup began by reviewing the inventory of AAPHD members’ areas of expertise from the organization’s membership renewal form. Those areas were then clustered by themes and transformed into seven draft research objectives. General topics covered by the objectives included policy and legislation, delivery system innovations, the DPH workforce, DPH education, psychosocial factors/social determinants of health, data science, and surveillance and epidemiology.

To solicit input on the draft objectives, the workgroup developed an online survey by using SurveyMonkey and distributed it via email to all AAPHD members (*n* = 503) in September 2021. Members were asked to: (1) indicate their level of agreement on whether the draft objectives reflected an important area of focus for DPH research moving forward; and (2) provide open-ended comments regarding wording suggestions or additional questions, topics, or methodologies for consideration within the objective. Close-ended response options included Strongly Agree, Agree, Disagree, Strongly Disagree, and Do not Know/Not Sure. The complete survey instrument is included in the online supplemental material.

## 3 | Results

A total of 115 AAPHD members responded to the survey (~23% response rate). For six of the seven objectives, more than 90% (*n* ≥ 103) of respondents agreed or strongly agreed that it reflected an important area of focus for DPH research. For the objective related to data science, 13% (*n* = 15) responded “don’t know/not sure.” The proportion of respondents that provided open-ended comments for the seven objectives ranged from 10% to 19% (*n* = 11–22).

Themes among member comments suggested three key revisions: (1) shortening the objective statements; (2) removing the example topic areas within the objectives; and (3) emphasizing the commonality of potential methodological approaches, study designs, and outcomes across many or all research areas. In response, the workgroup transformed the objectives into *Key Research Areas* and restructured methodological approaches and outcomes to cut across all these areas.

## 4 | AAPHD 5-Year Research Agenda

This research agenda proposes six key areas for research in the DPH field:

1. Oral Health Policy and Legislation
2. Delivery System Innovations and Care Models
3. Oral Health Workforce
4. Dental Public Health Education
5. Social Determinants of Health
6. Epidemiology and Surveillance Systems

**FIGURE 1 |:**
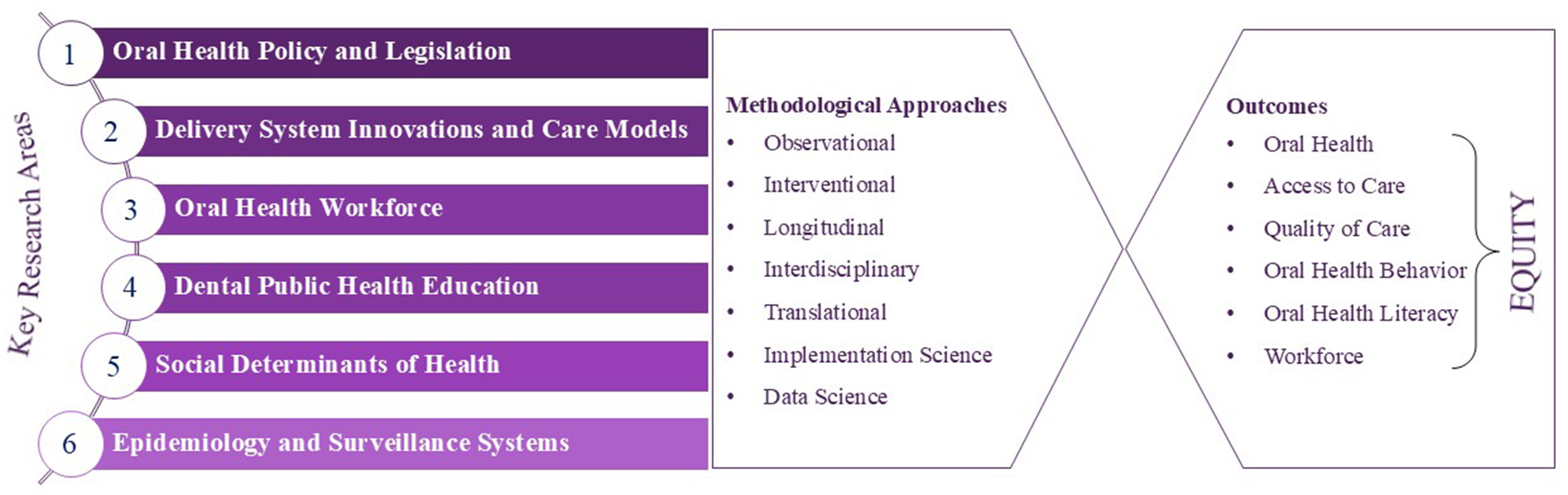
The 2025 American Association of Public Health Dentistry (AAPHD) research agenda—Key research areas, methodological approaches, and outcomes. [Color figure can be viewed at wileyonlinelibrary.com ]

Figure 1 presents six key research areas, cross-cutting methodological approaches, and oral health outcomes.

The following sections elaborate on priority topics, research questions, areas where evidence is limited for each of the key research domains, and describe existing and emerging methodological approaches and their application in studying various oral health outcomes.

## 5 | Key Research Area # 1—Oral Health Policy and Legislation

Policy interventions and legislation are significant in creating an environment where all individuals have equal opportunities to live healthy lives and prosper. The DPH field supports oral health policy that helps prevent oral diseases and promote equitable oral and overall health. Conducting research to assess the effectiveness of oral health policy and legislation is critical in understanding the efficacy of policies in improving the population’s oral and overall health. Research in this area focuses on the evaluation of various health policies, laws, regulations, procedures, administrative actions, incentives, or voluntary practices of governments and other institutions that could impact population oral and overall health. Understanding the short-and long-term outcomes of policies is necessary to guide policymakers, advocates, and stakeholders to improve and develop mechanisms to sustain, revise, or strengthen effective policies. Therefore, rigorous evaluation and research on these policies are critical for deciphering their effects on the community’s oral health and overall health.

During the past two decades, health services and health policy research in oral health have grown significantly. Research on major oral health policies and laws, such as dental coverage for children and adults enrolled in Medicaid and seniors in Medicare, has provided evidence to better understand the impact of these state-and national-level programs. For example, studies examining trends in Medicaid enrollment and dental utilization among children have shown major progress in dental care access for this population [7, 8]. However, the absence of uniformity of adult dental benefits in Medicaid coupled with selective expansion of Medicaid under the Affordable Care Act has led to important scholarly contributions about the detrimental effects of this state-level heterogeneity on dental care access for adults and seniors with low income [9].

While assessing policies’ impact on improving access and utilization of oral health care is crucial, it is equally essential to evaluate their broader effects on overall health and quality of life. Those outcomes may include determining the impact of Medicaid dental coverage on chronic disease management, such as better diabetes control, and the effects of policies on social and economic outcomes, such as job satisfaction among adults, or measuring the impact of extending post-partum Medicaid eligibility for pregnant people on their workforce entry/reentry.

Research on topics that can help inform the development of new policies to support oral health and overall health or changes in laws and policies to enable healthy behaviors is essential. Such policies might include consumer-friendly food labeling; taxation and cost of sugar-sweetened beverages, cigarettes, and alcohol; programs that promote healthy food and lifestyle choices; clean and fluoridated water initiatives; smoke-free laws; policies and regulations on food safety and advertising; and school health programs. Additionally, policies that encourage vaccinations and screenings, such as oral cancer and HPV, should be studied for their long-term impact [10–12].

The connection between policy and health is well established. Robust research in this area is vital to further strengthen existing laws and policies, create new ones, and identify and implement effective programs to promote population oral health and to support their overall health and well-being.

## 6 | Key Research Area # 2—Delivery System Innovations and Care Models

Components of the dental care delivery system in the United States (US) include providers, practice settings and systems, and payment models [12]. At a macro level, it is critical to understand the impacts of these three interrelated components on the domains of access, quality, and oral health outcomes, as well as equity within each of those domains. There is a need for research to test the effectiveness of innovative pilot and demonstration projects that aim to improve access and quality of care. Promising models could then be scaled up to larger community-based research projects. Improvements in scientific knowledge about the structure, organization, and performance of the oral health delivery system will help inform, develop, and scale evidence-based policy decisions, training programs, and care delivery models. The 2021 *Oral Health in America Report* published by the National Institute of Dental and Craniofacial Research (NIDCR) underscored key priorities related to oral health care delivery in the US, including improving prevention and upstream factors, leveraging and understanding the oral-systemic relationship, and understanding the financial linkages between oral-systemic health and insurance benefits [13].

Priority topics within this key area of research include provider-related factors such as changing practice patterns of the dentist workforce and integration with non-dental providers; practice settings and systems, such as medical-dental integration in dental care delivery and financing, emergency department diversion programs, and telehealth; payment models, including alignment and growth of quality measurement and improvement in dentistry to test the effectiveness of value-based payment arrangements. Many of these topics lack sufficient quality research regarding their potential impact on access, quality, and outcomes in oral health. For example, despite considerable scholarly attention given to understanding the evolving practice patterns of the dentist workforce—such as the decline in rural practice locations and ownership, and the rising trend toward larger group, corporate/Dental Service Organization (DSO) practices, and the role of private equity—the impacts of these evolving trends on access to dental care are only beginning to be studied.

Scholarship in practice settings and systems is far-ranging and generally focused on improving access and efficiency, and reducing the cost of dental care delivery. One area of considerable scholarly progress is the substantial evidence on medical professionals’ role in providing preventive oral health services and anticipatory guidance [14–19]. Important areas of research opportunity include additional medical-dental integration models such as embedding dental personnel into medical care delivery settings or expanding the small body of evidence on the cost-effectiveness of embedding dental benefits into health plans for people with chronic conditions [20–23].

The use of telehealth exponentially increased during the COVID-19 pandemic, yet much of the scholarly work on teledentistry has focused on diagnostic accuracy, provider and patient perceptions, and implementation feasibility [24]. There is a great need to understand the degree to which synchronous and asynchronous teledentistry models can improve access and efficiency of dental care, particularly for those with geographic, transportation, or other major barriers to care.

Although there has been significant discussion in the oral health community about the need for alternative payment models that incentivize value over volume, there have been very few examples of the actual use of value-based care or pay-for-performance in dental care delivery. Thus, partnerships are needed between researchers and the few organizations that have implemented these models to understand their effects and, if promising, promote scalability. Underpinning value-based care requires additional scholarship and development on quality measurement and improvement in dentistry, particularly including patient-reported experience and outcome measures.

Research in this domain is crucial to advance our scientific understanding of the impacts of promising models and organizations and effectively promote the expansion of models that demonstrate improvement in access, quality, and oral health outcomes.

## 7 | Key Research Area # 3—Oral Health Workforce

A competent, well-trained, diverse, and adequately sized workforce is the foundation for maintaining and improving population oral health. More than 55 million Americans live in dental health professional shortage areas [25]. Evidence shows that living in communities with limited access to oral health professionals can lead to poorer oral health outcomes [26]. Some common workforce-related access barriers include limited provider participation in public programs such as Medicaid, geographic maldistribution of dental providers, low dental workforce diversity, provider reimbursement challenges, lack of appointment availability, state variation in scope of practice laws, and licensing barriers for internationally educated dentists [27–29].

The traditional dental workforce model of dentists, dental hygienists, and dental assistants has evolved to address barriers hindering optimal oral health, especially among those who need the most care. For example, dental therapists are mid-level dental providers who can help expand the reach of dental services, especially to underserved and at-risk populations. As of November 2024, dental therapists are authorized to practice in 14 states: Alaska, Arizona, Colorado, Connecticut, Idaho, Maine, Michigan, Minnesota, Nevada, New Mexico, Oregon, Vermont, Washington, and Wisconsin [30]. Although there are limited studies on the benefits of this workforce model for access to care in the US, emerging evidence from Minnesota and Alaska suggests improvements in access in these early adopter states [31–34]. Nevertheless, more research is needed to fully understand the impact of this workforce model as additional states pass legislation allowing dental therapists to practice.

Dentistry has also engaged non-dental members of the healthcare workforce, including primary care providers (PCPs), community health workers, school nurses, and other oral health champions to promote oral health awareness and increase oral health literacy. However, the effects of many team-based care models are just beginning to be studied. More research is crucial to develop and assess new and existing models of team-based care approaches to support advocacy and scale those proven to be most effective.

Diversity in the dental workforce is another critical issue. The racial and ethnic composition of the oral health workforce does not mirror the population it serves—particularly for Black and African American, Hispanic/Latino/a/x/e, and American Indian/Alaska Native communities [35, 36]. Various programs and policies have been developed to increase the number of dentists from historically underrepresented communities, such as pathway programs, but their effectiveness is not well investigated. Additionally, the available evidence is mixed regarding the impact of patient-provider concordance on patient experience and health outcomes in the medical literature [37] and more research is needed in dentistry to understand the most effective approaches to improve patient experiences with care.

Studies have examined various workforce-related questions, yet there is an ongoing need to pose new questions and decipher them to improve population oral health. Examples of research topics include exploring how an integrated workforce can enhance efficiency, assessing reimbursement models to sustain and expand the oral health workforce in remote areas, evaluating pathways for internationally-educated dentists to enter the US oral health workforce, and examining patterns of workforce growth and attrition.

Additional workforce-related priority research areas identified in the 2021 NIDCR *Oral Health in America Report* include examining scope of practice laws and their impact on the efficacy and efficiency of care delivery, as well as engagement of the workforce in public programs such as Medicaid and Medicare [13].

The oral healthcare workforce is inextricably related to access and utilization of health services. Examining challenging research questions on workforce issues and acting on findings are fundamental to reforming and creating a sustainable and thriving oral health workforce that effectively reduces disparities and efficiently enhances oral health outcomes.

## 8 | Key Research Area # 4—Dental Public Health Education

DPH education is crucial in creating a knowledgeable, high-performing, and culturally sensitive dental and DPH workforce [38]. Conducting research studies on DPH education could help identify gaps, enhance curriculum, improve teaching methods, and assess its impact on care quality and patient outcomes. DPH education is delivered at both the predoctoral and postdoctoral levels. In predoctoral education, the DPH content varies significantly among US dental schools aiming to familiarize students with dental practice in underserved communities after graduation. Dental students are often introduced to some DPH topics such as disease prevention, behavioral dentistry, community dentistry, evidence-based dentistry, workforce and scope of practice, community outreach, oral health literacy, cultural sensitivity, and social determinants of health [39]. Existing research on the value and impact of DPH courses and extramural experiences in underserved communities in predoctoral programs has explored factors that influence graduates to pursue DPH residency or work in underserved areas, offering insights into the impact of exposure to DPH concepts and clinical experiences [40]. However, future research is needed to assess the impact of community dentistry courses within predoctoral curricula on student learning and their contributions to community oral health practices. Additionally, research could explore how early exposure to DPH in the predoctoral curriculum benefits students’ clinical skills, confidence, and readiness for patient care. Moreover, studies could assess the impact of new private dental schools on post-graduation career paths and examine the overall effectiveness of these initiatives.

At the postdoctoral level, DPH residency programs have well-defined objectives aimed at providing education following the standards set by the Commission on Dental Accreditation (CODA) [38]. Additionally, these programs are designed to ensure that postgraduate students achieve the 10 core DPH competencies, which encompass a broad range of skills such as oral health program management and leadership, designing surveillance systems, (oral) health policy, community-based care, and integrating the social determinants of health into DPH practice [41]. The primary goal of the residency programs is to equip DPH postgraduate students with the essential knowledge and skills necessary to lead and manage oral health programs across diverse sectors, including local, state, national, and private domains [41]. These comprehensive educational programs are structured to prepare DPH residents for practical challenges they may encounter in the field, placing a specific emphasis on addressing the oral health needs of underserved populations. By a combination of didactic courses, hands-on field experience, and research activities, and a holistic approach [42], DPH residents will be well-prepared to navigate the complexities of their roles and contribute effectively to oral health initiatives.

Research at the postdoctoral level could explore the impact of various educational components—such as structure, course content, program length, and ABDPH board certification—on identifying shortcomings and gaps in current training programs. Priority topics include exploring gaps, evaluating the effectiveness of postgraduate education in enhancing residents’ competencies, and assessing the practical application and relevance of core competencies in the daily responsibilities of DPH professionals. According to limited research, DPH trainees, particularly foreign-trained dentists, face training challenges during and after their postgraduate residency programs, highlighting opportunities to strengthen DPH programs to build a competent workforce [43]. Another study suggested including courses to equip graduates with skills valued in the job market, ensuring graduates are well positioned [44]. Additionally, research showed that diplomates are concentrated in academia, indicating a need to update competencies and encourage nonacademic career paths for DPH graduates to maintain the field’s impact [45].

Furthermore, research is needed to understand the perspectives of program directors, key DPH stakeholders, and funders at the national level. Such insights could inform the development of a comprehensive roadmap and robust curriculum designed to cultivate a workforce that is competent, adaptable, and focused on the population’s oral health and patient-centered dental care. This research would not only improve community oral health but also ensure appropriate employment opportunities for DPH professionals.

Additionally, uncovering factors influencing the quality of DPH education and its impact on oral health outcomes is crucial. This research can optimize the dental workforce and improve dental care services for diverse communities, ensuring DPH professionals are well-prepared and equipped to address complex oral health.

## 9 | Key Research Area # 5—Social Determinants of Health

The World Health Organization (WHO) defines social determinants of health (SDOH) as the conditions in which people are born, live, learn, work, play, worship, and age that affect health and quality of life [46]. These conditions have been recognized as the upstream factors that impact health outcomes by shaping the degree of opportunity to engage in healthy behaviors and structuring power and resources. The five broad categories of SDOH outlined in Healthy People 2030 are Economic Stability, Education Access and Quality, Health Care Access and Quality, Neighborhood and Built Environment, and Social and Community Context [46]. Each broad area includes many specific determinants likely to impact health and oral health. While some scholarly work has examined oral health-contributory factors at multiple levels together, including neighborhood and system levels, most studies have focused on a particular social determinant such as social capital, food access, or transportation barriers. More research is needed to understand and quantify the mechanisms connecting SDOH to oral health, including mediating and moderating factors. Though our understanding and conceptualization of the social determinants of health has increased substantially in recent decades, there is a need to develop and update conceptual frameworks on SDOH and oral health that can be used as theoretical foundations to center SDOH scholarship on oral health.

While intervention research on SDOH is necessarily complex and difficult, one area of opportunity is to examine the impact of interventions that provide health-related social needs (HRSN), such as healthy food prescriptions or transportation support for healthcare appointments. The United States Department of Health and Human Services approved two state Medicaid program waivers in 2022 that will implement HRSN interventions for special populations [47], and the evaluation and scholarly work coming out of this policy experiment would benefit from including the effects of these interventions on oral health outcomes.

A critically important area for which there is an emerging body of evidence is the role of structural racism as a social determinant of health on oral health outcomes. Though there is no consensus about whether structural racism is a driver of SDOH or a SDOH in and of itself, structural racism is certainly known to inequitably structure the conditions in which we live, grow, work, and age. Most recent scholarship on racism and oral health has examined racism at an individual level, and more research is needed to understand the connection between racism at the structural level and oral health access and outcomes [48, 49].

SDOH are the root causes that create inequitable conditions that facilitate or hinder good oral health by race, income, ability, gender identity, and others. Understanding, intervening, and advocating for change in these root causes are critical to moving toward oral health equity.

## 10 | Key Research Area # 6—Epidemiology and Surveillance Systems

The field of oral epidemiology focuses on conditions related to oral diseases, including dental caries, periodontal problems, malocclusion, temporomandibular joint disorders, dental hypersensitivity, toothache, orofacial disorders, edentulism, oral and pharyngeal cancers, hard and soft tissue diseases, and traumatic injuries to the face, mouth, teeth, and gums. These disorders have physical, psychological, social, and financial impacts, making it crucial for clinicians and public health experts to understand them and find ways to prevent or manage them. The definition of oral health highlights the importance of oral health and its impact on overall health. According to the International Dental Federation (FDI), oral health refers to “the ability to speak, smile, smell, taste, touch, chew, swallow and convey a range of emotions through facial expressions with confidence and without pain, discomfort and disease of the craniofacial complex (head, face, and oral cavity)” [50]. This definition underscores the essential functions of the oral cavity for quality of life and mental health, which are still poorly understood.

Oral health has three major attributes: it is a fundamental component of physical and mental health, plays an essential role in the quality of life, and involves being free of pain and discomfort. It is essential to understand these attributes at the population level, such as how oral conditions affect the physical and mental health of different populations. However, the distributions and types of different physical and mental disorders due to poor oral health have not yet been thoroughly investigated. Additionally, reliable diagnostic tools to study these impacts are crucial. Oral epidemiology provides clinicians and public health experts with the tools and strategies for a deeper understanding of oral diseases, their risk factors, populations at risk, diagnostic tools, intervention strategies, and planning and evaluating oral health care services and their impact on quality of life.

Prioritizing research in this field can significantly expand our knowledge in the design of targeted oral health plans, particularly for marginalized communities that experience higher rates of oral health problems. By conducting research at national, state, and community levels, researchers can pinpoint high-risk populations and geographic areas that require targeted interventions and public health strategies. Furthermore, epidemiologic studies help in assessing the effectiveness of these interventions over time, ensuring that resources are allocated efficiently and policies are adapted to meet evolving needs. Using tools and methodologies of (oral) epidemiology can lead to improved oral health outcomes and better quality of life for individuals and communities.

Some key research topics include: (a) Providing accurate and up-to-date data on the incidence, prevalence, and associated risk factors of these conditions across diverse populations and subgroups, at global, national, and local levels. Such data are essential for examining primary, secondary, and tertiary preventive measures, as well as for shaping policies and addressing workforce concerns effectively; (b) Creating and developing reliable surveillance tools to identify individuals at elevated risk for oral diseases/conditions and their related risk factors. Those tools are essential for targeted resource allocation and more efficient utilization; (c) Developing research on diagnostic codes applicable to administrative data, such as insurance records, to enable an additional method of disease surveillance and linking care patterns to oral health outcomes [51]; (d) Establishing reliable, evidence-based screening criteria is pivotal to assessing the oral health needs of populations and implementing effective screening programs; (e) Conducting well-designed epidemiological studies exploring the association between systemic and oral diseases; (f) Exploring the use of technology in epidemiological studies, such as integrating modern tools like big data, machine learning, Geographic Information System (GIS), and wearable devices to enhance data collection, analysis, and interpretation which allow for more precise tracking of disease patterns, identification of risk factors, and real-time monitoring of health trends [52].

## 11 | Sample Research Topics and Questions Aligned With Proposed Key Research Areas

Table 1 provides example research topics and questions within each priority topic, to direct researchers’ attention toward gaps and shortcomings of current evidence. It is essential to acknowledge that these examples are not exhaustive, and there exists a vast array of topics and research questions that can be formulated beyond what is listed in this table. Additionally, there are many areas in which there is conceptual overlap; thus, some research questions may address multiple priority topics. The table serves as a starting point, offering the authors’ recommendations for potential avenues of exploration.

## 12 | Methodological Approaches

A variety of research methods and study designs are used in DPH research to assess different topics, including observational and experimental study designs, interdisciplinary approaches, implementation science strategies for translating findings into practice, leveraging data science principles including using large datasets, and employing analytical techniques to make causal inferences.

Observational studies are a crucial methodological approach in DPH research. These studies, categorized as prospective or retrospective, include cross-sectional, cohort, and case–control designs. *Prospective studies* involve following a group of individuals over a period to determine disease occurrence or intervention impact. *Retrospective studies*, conversely, analyze data retrospectively to determine if there is an association between exposure and disease outcomes. *Cross-sectional studies*, a common approach in DPH research, provide a snapshot of the population at a given time, such as the prevalence of oral diseases and their associated risk factors in specific communities at a specific timeframe. *Cohort studies* follow a group of individuals longitudinally to determine disease incidence or exposure effects, while *case–control studies* compare individuals with a specific disease to those without, identifying pertinent risk factors.

Experimental and particularly randomized controlled trials stand as the gold standard for establishing causality in (dental) public health research. These studies entail the random assignment of participants to either an intervention or control group to assess the intervention’s effect on the outcome of interest. Alternatively, in cases where randomization is not possible, quasi-experimental research using econometric analytic methods leverages natural experiments such as policy changes to try to establish a causal impact. Additionally, emerging study designs, such as step-wedge [53] or SMART [54] (Sequential, Multiple Assignment, Randomized Trials) trials, offer valuable avenues for gaining further insights.

Team Science is essential in DPH research. Collaborative efforts across dentistry, medicine, behavioral health, business, economics, sociology, engineering, and others are crucial for understanding the multifaceted nature of oral diseases and formulating effective interventions. Broader collaborations can yield valuable insights into behavior change, organizational change, and systems thinking. Working collectively in a team allows researchers to pool their knowledge and skills, addressing complex problems that cannot be resolved by any single individual or field alone. Bringing together researchers with diverse backgrounds and perspectives generates novel ideas and approaches that might not be achievable within traditional disciplinary silos. Additionally, involving multiple researchers with different skill sets and perspectives, Team Science can reduce the likelihood of errors or biases that can occur in individual studies [55].

Implementation Science—translation of science into practice— is another critical aspect of DPH research. Of interest to the DPH approach, three key stages are noteworthy: (1) translation into dental and medical practice; (2) translation into population settings; and (3) translation into policy. These stages would benefit significantly from an implementation science approach, defined as “methods to promote the systematic uptake of clinical research findings and other evidence-based practices into routine practice and hence improve the quality and effectiveness of health care” [56].

In DPH research, using large datasets and data science techniques to make causal inferences is becoming increasingly important. Big data, often derived from insurance claims and electronic health records, is at the forefront of data science. In recent years, Machine Learning (ML) has enabled researchers to uncover complex patterns in big datasets, making it highly effective in identifying trends, predicting outcomes, and uncovering hidden relationships in large datasets [57]. However, it also faces challenges in establishing causal relationships, as it often relies on associations rather than direct cause-and-effect evidence. Despite these challenges, ML can be a powerful tool in DPH research for informing predictive models and optimizing interventions.

Furthermore, there are opportunities to integrate additional large datasets, which can enhance the diversity of the population included, addressing concerns about generalizability. For example, combining data from dental insurance claims with demographic information from national health surveys (such as the National Health and Nutrition Examination Survey, NHANES) could provide a broader, more representative sample of different socioeconomic groups, geographic locations, and racial/ethnic backgrounds. This integration would help ensure that the findings are inclusive and applicable to a wider range of populations, reducing the bias that can arise from using a limited dataset. Because data science largely employs observational research approaches to derive insights, determining causation remains a challenge. Nevertheless, various techniques, such as quasi-experimental designs, differences-in-differences analyses [58] or propensity score matching can be used [59, 60].

## 13 | Oral Health Outcomes

**FIGURE 2 |:**
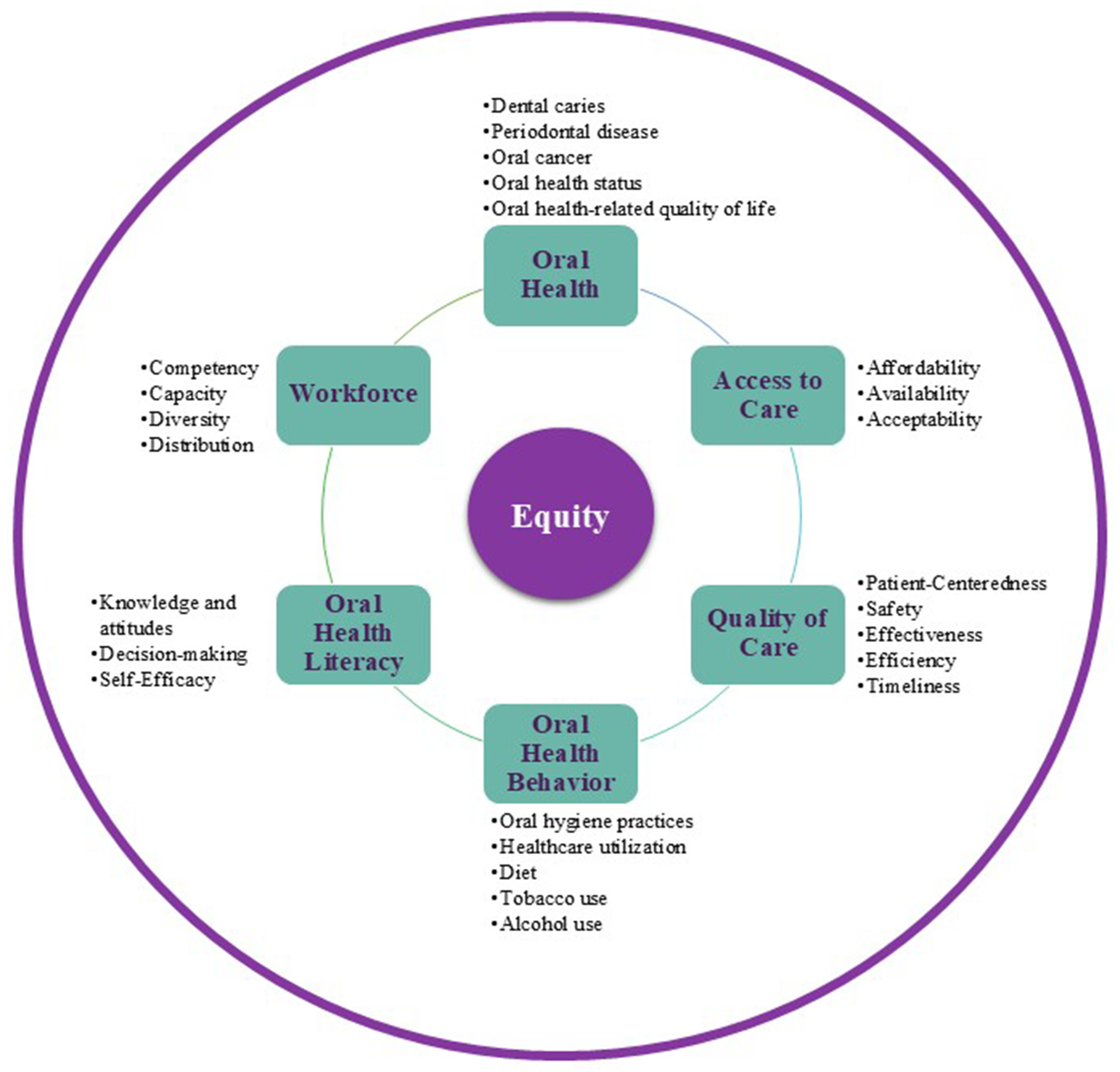
Domains of oral health outcomes and example measures within each domain. [Color figure can be viewed at wileyonlinelibrary.com]

The ultimate goal of all oral health-related research should be to advance the scientific understanding required to improve population oral health and achieve oral health equity. The cross-cutting outcomes highlighted in this agenda span six domains, which represent oral health outcomes and/or the downstream, most proximal predictors of oral health: *oral health, access to care, quality of care, oral health behavior, oral health literacy, and workforce, with equity cutting across each of the domains*. Figure 2 provides examples of more granular outcome measures within each of these domains. Future oral health-related research must move toward the goal of addressing equity in each of these domains. Broad-based improvement in oral health outcomes risks maintaining existing disparities if equity is not the explicit objective.

## 14 | Discussion and Conclusions

This DPH research agenda includes six broad areas of research and highlights their significance, progress in their body of evidence, and potential opportunities to address research gaps. This research agenda provides a framework for new and seasoned researchers to consider as they formulate their research questions. Its overarching goal is to stimulate high-quality research that can be translated into practical applications and ultimately address oral health challenges and promote well-being at the population level.

The research agenda may also be used to influence policy and shape community programs, tailor interventions to specific populations, and address their unique oral health needs. The evolution of the DPH field is evident in its commitment to applying research findings and exploring future directions to meet changing population needs, enhance preventive efforts, and contribute to overall health and well-being.

The AAPHD aims to regularly measure progress in these key areas, committing to conducting environmental scans and updating the research agenda to ensure it remains current and relevant. Regular updates will enable the integration of new research findings, emerging technologies, shifts in DPH priorities, and evolving DPH trends. This process ensures that the agenda remains a relevant and effective guide for efforts to enhance community oral health and achieve oral health equity.

The AAPHD encourages DPH partners, including federal agencies, related DPH organizations and foundations, and dental associations, to continue to advocate for, support, use, and fund high-quality DPH research, to translate emerging evidence into improvement in oral health and oral health equity.

## Supplementary Material

Supplemental Material-Survey

Additional supporting information can be found online in the Supporting Information section. **Data S1:** Supporting Information.

## Figures and Tables

**TABLE 1 |:** Research agenda key areas, priority topics, and example research questions.

Priority topics	Example research questions
Key research area #1—Oral health policy and legislation	
Public and commercial dental coverage	How do cost-sharing mechanisms such as copays and annual benefit maximums affect access to dental care for people enrolled in Medicaid?
Medicare policy and regulation	What would be the impact of providing comprehensive dental coverage in Medicare on the improvement of chronic disease outcomes?
Sugar tax	To what degree does the implementation of a soda tax reduce dental caries?
Vaping regulation	How does implementing vaping regulations impact accessibility and consumption of e-cigarettes, and subsequently influence oral health conditions?
Community water fluoridation	How does the preserve of fluoride in drinking water interact with other environmental factors (e.g., diet, socioeconomic status) in influencing oral health outcomes?
Scope of practice regulations	What is the impact of dental hygienists being allowed to provide preventive services without a prior exam by a dentist on receipt of preventive services?
Key research area #2—Delivery system innovations and care models	
Value-dased/pay-for-performance models	What is the impact of a value-based payment model versus fee-for-servlce payment on various aspects of dental practice and the utilization of dental service, preventive care, and oral health outcomes?
Medical dental integration	What is the impact of dental care provided in alternative care settings on access to care and oral health outcomes?
School health programs	What trends are occurring in the number and focus of school-based oral health programs?
Quality measurement and improvement	To what extent do quality measurement and improvement initiatives improve access and quality of dental care?
Telehealth	What are the impacts of various synchronous and asynchronous tele dentistry models on access to care?
Minimally invasive dentistry	What are the effects of SDF and other minimally invasive therapies on root caries among older adults?
Key research area #3—Oral health workforce	
Workforce composition	What are the dental workforce needs at the regional, state, and local levels? (Simulation or forecasting analyses)
Geographic distribution and availability	What are the impacts of national and a state loan repayment programs on dentist practice characteristics such as practice location and provision of care to underserved population?
Pathway programs	What are the effects of pathway programs on dental workforce diversity?
Workforce diversity	What approaches are most effective in supporting the recruitment and retention of racially minoritized professionals in dental academic faculty positions?
Community health workers	Do community health worker models improve oral health behaviors and dental care utilization?
Dental therapists	What is the impact of the dental therapist workforce on access, quality, and cost of oral health care?
Key research area #4—Dental public heath education	
Predoctoral DPH-related education	What predoctoral education factors are associated with dentist and dental hygienist service to marginalized populations post-graduation?
postdoctoral students’ job prospects	What are the most common job prospects and what skills are required to be recruited in relevant DPH organization?
Career paths and recruitment to DPH	What factors lead dental school graduates to pursue the specialty of DPH?
Retention of DPH graduates in the field	How do educational programs and partnerships influence the development of DPH capacity and job prospects at national, state, and local levels? What barriers hinder the development of DPH capacity and job prospects, and how can these be overcome at national, slate, and local levels? What factors contribute to DPH graduates leaving the DPH workforce?
Key research area #5—Social determinants of health	
Social determinants of health and oral health	How can neighborhood safety affect oral health outcomes?
Health-related social needs (HRSN)	What is the impact of HRSN interventions on oral health?
Systemic racism and oral health	What are the mechanisms that connect systemic racism and oral health?
Key research area #6—Epidemiology and surveillance systems	
Surveillance system	Are there new measures of oral health or associated factors that require adjustments to national surveillance programs?
Oral health disparities and inequities	For which populations have oral health inequities improved, worsened, or stayed the same?
Electronic health records	What is the impact of interoperable electronic medical and dental records on referrals from medical to dental care?
Oral health-related behaviors	What would be the impact of aligning payment systems to incentivize evidence-based behavior change interventions such as motivational interviewing?
Oral health and systemic diseases	What are the relationships between oral health and chronic systemic diseases?
Use of technology in epidemiological Studies	What are the evidence-based practices for the use of technology (e.g., imaging, teledentistry) in epidemiological studies?

## Data Availability

The data that support the findings of this study are available on request from the corresponding author. The data are not publicly available due to privacy or ethical restrictions.
