# Supplementary material for: Forward Outlook on Oral Health: The American Association of Public Health Dentistry’s 2025 Research Agenda for Dental Public Health
Source: J Public Health Dent. Author manuscript; Available in PMC 2026 May 4. (PMC13138120; doi:10.1111/jphd.70005)
Supplement: Supplemental Material-Survey [file NIHMS2163977-supplement-Supplemental_Material-Survey.docx]

**AAPHD Research Agenda Supplemental material**

Survey instrument:

**Content in the body of email message**

Subject line: “Participation requested: Development of Dental Public Health Research Agenda”

Body of message:

Dear AAPHD member,

On behalf of AAPHD, the Council on Scientific Information seeks member feedback to develop a research agenda to guide research in the field of dental public health for the next 5 years. Member responses will help to prioritize objectives and identify specific research questions and methodologies that should be included in the agenda. We are interested in input from all members, whether they conduct or utilize DPH-related research.

This survey will take approximately 10 minutes to complete. The link to the survey is below.

<insert link>

Thank you in advance for your participation!

Members of the Council on Scientific Information

**Survey content**

Thank you for contributing to the development of AAPHD’s Dental Public Health Research Agenda. Below are seven proposed broad objectives, drafted by the members of the Council on Scientific Information, to guide research in the field of dental public health over the next 5 years. For each objective:

1. Indicate your level of agreement about whether the objective reflects an important area of focus for DPH research moving forward.
2. Provide comments regarding:
   1. Wording suggestions or clarifications on the objective itself
   2. Specific research questions, topics, or methodologies that are needed within each objective.

| **Objective** | **Strongly agree** | **Agree** | **Disagree** | **Strongly disagree** | **Do not know/Not sure** | **Comments** |
| --- | --- | --- | --- | --- | --- | --- |
| 1. Evaluate the impact of oral health policy and legislation, such as commercial and public dental insurance coverage, sugar or tobacco policy and taxes, and community water fluoridation on population oral health | O | O | O | O | O | <open text field> |
| 1. Leverage principles of a learning healthcare system – including quality improvement, measurement, and implementation science – to examine the impact of delivery system innovations such as alternative payment models, alternative care settings/modalities, and interprofessional practice, on improving access to care, reducing inequities, and improving oral health outcomes. | O | O | O | O | O | <open text field> |
| 1. Examine the impact of all oral health workforce models – including distribution, diversity, and state regulations – on access to care, outcomes, and costs. | O | O | O | O | O | <open text field> |
| 1. Evaluate and evolve DPH education and infrastructure to develop a diverse, culturally competent workforce enabled to meet the oral health needs of the public. | O | O | O | O | O | <open text field> |
| 1. Conduct interdisciplinary research on the impact of psychosocial factors, such as social determinants of health, cultural competency, health literacy, equity, and systemic racism, on oral health. | O | O | O | O | O | <open text field> |
| 1. Leverage principles of data science, artificial intelligence, machine learning, and natural language processing to enhance dental public health research. | O | O | O | O | O | <open text field> |
| 1. Conduct surveillance and epidemiological research to understand 1) the burden of, and risk factors for, oral diseases, including caries, periodontal disease, oral & oropharyngeal cancer; 2) the impact that oral health has on systemic conditions, and vice versa; 3) the impact of preventive measures such as community water fluoridation, sealants, fluoride varnish; 4) the impact of minimally invasive dentistry, such as SDF, across the lifespan and across populations. | O | O | O | O |  | <open text field> |

1. In considering all of these objectives collectively, please share any additional objectives that you think should be included as part of the research agenda or any general comments.

<open-text field>

1. How long have you have been a member of AAPHD?

- Less than a year
- 1-2 years
- 3-4 years
- 5 years or more
- Don’t know

1. What is your occupation?
2. If you are involved in conducting research, what are your three main areas of expertise/interest?
   1. __________
   2. __________
   3. __________
   4. N/A – I do not conduct research
